# Understanding Home Hemodialysis Patient Attrition: A Cohort Study

**DOI:** 10.1177/20543581211022195

**Published:** 2021-06-13

**Authors:** Bailey Paterson, Danielle E. Fox, Chel Hee Lee, Victoria Riehl-Tonn, Elena Qirzaji, Rob Quinn, David Ward, Jennifer M. MacRae

**Affiliations:** 1 Cumming School of Medicine, University of Calgary, AB, Canada; 2 Department of Community Health Sciences, University of Calgary, AB, Canada; 3 Department of Mathematics and Statistics, University of Calgary, AB, Canada; 4 Division of Nephrology, Cumming School of Medicine, University of Calgary, AB, Canada; 5 Department of Cardiac Sciences, University of Calgary, AB, Canada

**Keywords:** home hemodialysis, technique failure, training failure

## Abstract

**Background:**

Home hemodialysis (HHD) offers a flexible, patient-centered modality for patients with kidney failure. Growth in HHD is achieved by increasing the number of patients starting HHD and reducing attrition with strategies to prevent the modifiable reasons for loss.

**Objective:**

Our primary objective was to describe a Canadian HHD population in terms of technique failure and time to exit from HHD in order to understand reasons for exit. Our secondary objectives include the following: (1) determining reasons for training failure, (2) reasons for early exit from HHD, and (3) timing of program exit.

**Design:**

A retrospective cohort study of incident adult HHD patients between January 1, 2013—June 30, 2020.

**Setting:**

Alberta Kidney Care South, AKC-S HHD program.

**Participants:**

Patients who started training for HHD in AKC-S.

**Methods:**

A retrospective, cohort study of incident adult HHD patients with primary outcome time on home hemodialysis, secondary outcomes include reason for train failure, time to and reasons for technique failure. Cox-proportional hazard model to determine associations between patient characteristics and technique failure. The cumulative probability of technique failure over time was reported using a competing risks model.

**Results:**

A total of 167 patients entered HHD. Training failure occurred in 20 (12%), at 3.1 [2.0, 5.5] weeks; these patients were older (*P* < .001) and had 2 or more comorbidities (*P* < .001). Reasons for HHD exit after training included transplant (35; 21%), death (8; 4.8%), and technique failure (24; 14.4%). Overall, the median time to HHD exit, was 23 months [11, 41] and the median time of technique failure was 17 months [8.9, 36]. Reasons for technique failure included: psychosocial reasons (37%) at a median time 8.9 months [7.7, 13], safety (12.5%) at 19 months [19, 36], and medical (37.5%) at 26 months [11, 50].

**Limitations:**

Small patient population with quality of data limited by the electronic-based medical record and non-standardized definitions of reasons for exit.

**Conclusions:**

Training failure is a particularly important source of patient loss. Reasons for exit differ according to duration on HHD. Early interventions aimed at reducing train failure and increasing psychosocial supports may help program growth.

## Abrégé

**Contexte:**

Pour les patients atteints d’insuffisance rénale, l’hémodialyse à domicile (HDD) offre une modalité flexible et centrée sur le patient. Une meilleure adhésion à l’HHD s’obtient en augmentant le nombre de patients initiés à cette modalité et en réduisant l’attrition grâce à des stratégies visant la prévention des causes modifiables d’abandon.

**Objectifs:**

Notre principal objectif était de décrire une population canadienne de patients suivant des traitements d’HDD en ce qui concerne l’échec de la modalité et de délai avant l’abandon de l’HDD, afin de comprendre les raisons qui mènent à cet abandon. En deuxième lieu, nous souhaitions: (1) déterminer les raisons de l’échec de la formation sur la modalité, (2) les raisons de l’abandon précoce de l’HDD et (3) le moment du retrait du programme.

**Méthodologie:**

Il s’agit d’une étude de cohorte rétrospective portant sur les patients adultes ayant adopté l’HDD comme modalité entre le 1er janvier 2013 et le 30 juin 2020.

**Cadre:**

Le programme d’HDD AKC-S (Alberta Kidney Care South).

**Sujets:**

Les patients ayant commencé une formation sur l’HDD avec le programme AKC-S.

**Méthodologie:**

Une étude de cohorte rétrospective portant sur les patients adultes traités par HDD ayant pour principal critère d’évaluation la période pendant laquelle la modalité a été adoptée par les patients. La raison de l’échec de la formation, le délai avant l’abandon de la modalité et les raisons de l’abandon ont constitué les critères d’évaluations secondaires. Un modèle de risques proportionnels de Cox a été employé pour déterminer les associations entre les caractéristiques des patients et l’abandon de la modalité. La probabilité cumulative d’abandon de la modalité au fil du temps a été rapportée à l’aide d’un modèle des risques concurrents.

**Résultats:**

Les résultats portent sur les 167 patients qui étaient passés à l’HDD. L’échec de la formation a été observé chez 20 patients (12 %) après 3,1 [2,0, 5,5] semaines; ces patients étaient plus âgés (*P* < .001) et présentaient au moins deux maladies concomitantes (*P* < .001). La transplantation (n = 35; 21 %), le décès (n = 8; 4,8 %) et l’échec de la technique (n = 24; 14,4 %) ont constitué les principales raisons d’abandon de l’HDD après la formation. Dans l’ensemble, le délai médian avant l’abandon de l’HDD était de 23 mois [11, 41] et le délai médian avant l’échec de la technique était de 17 mois [8,9, 36]. Des raisons psychosociales (37 %) après un délai médian de 8,9 mois [7,7, 13], l’innocuité (12,5 %) après 19 mois [19, 36] et des raisons médicales (37,5 %) après 26 mois [11, 50] ont expliqué l’échec de la technique.

**Limites:**

L’étude porte sur un faible échantillon de patients dont la qualité des données était limitée par le dossier médical électronique. Des définitions non normalisées des raisons de l’abandon limitent également les résultats.

**Conclusion:**

L’échec de la formation est un facteur qui joue un rôle particulièrement important dans l’abandon de l’HDD par les patients. Les raisons de cet abandon varient en fonction de la durée d’utilisation de la modalité. Des interventions précoces visant à réduire l’échec de la formation et à augmenter le soutien psychosocial pourraient aider à accroître l’adhésion au programme.

## What was known before

- It is difficult to grow a home hemodialysis (HHD) program due to frequent patient exit
- Training failure is a challenge for most programs
- There may be potentially modifiable reasons for technique failure and the subsequent exit to conventional hemodialysis

## What this adds

- Training failure may be associated with age and a higher degree of comorbidity
- Training time appears longer for patients who have technique failure due to psychosocial causes
- Most common reasons for technique failure include medical (38%) and psychosocial (21%) with earlier exit for psychosocial (median time 8.9 months) and later exit for medical concerns (26 months)

## What impact this may have on practice or policy

- Time to exit is an important metric for HHD programs
- Ealy intervention with enhanced psychosocial supports may modify the risk for technique failure

## Introduction

As the number of patients requiring dialysis continues to grow, the majority will pursue in-center conventional hemodialysis (ICHD) despite the fact that numerous benefits exist^1-3^ for home dialysis from both a patient centered and health-care policymaker standpoint. From a patient perspective, home hemodialysis (HHD) has the potential to improve quality of life (QOL),^2-4^ and provide clinical benefits due to the ease of which patients can tailor their dialysis regimen for increased frequency and/or duration as compared to ICHD. The freedom and flexibility provided by dialysis at home is an attractive draw for patients when deciding their modality.^5^

While transplant is the most cost effective form of renal replacement therapy, not all patients are suitable candidates. For those patients that are, the wait list is typically many years. Therefore for patients who are on dialysis there is a clear economic advantage associated with HHD over ICHD,^6-9^ and as a result, provincial Kidney Agencies across Canada have encouraged a nation-wide initiative to increase the number of patients that pursue home modalities. Over the past 15 years, the number of patients on HHD has increased but, despite this, the number of patients exiting the program has also increased^10^ resulting in overall limited program growth.^11^ Due to the significant resources used for HHD training, it takes anywhere from 9 to 12.6 months on therapy before cost savings are reached^12,13^ compared to ICHD. This makes the time to exit, and the reasons for exit from HHD an important metric for kidney programs but it is not widely reported in HHD studies. Unfortunately, the reasons behind HHD program exit remain unclear, specific contributing factors are often not documented^14-16^ and there is no data on whether the reasons for exit change over time. Identifying the reasons for exit from HHD and how they may change over time during the course of therapy, is an important first step in developing strategies that could prevent modifiable technique failure.

The main objective of this study is to explore the reasons behind patient exit from HHD in the Alberta Kidney Care South (AKC-S) program. Understanding reasons why patients leave HHD and the timing of these events will facilitate the development of targeted interventions and effective resource allocation to reduce modifiable reasons for patient exit. This knowledge will also be useful to understand the high patient turnover in HHD and allow the program to make accurate growth forecasts. Secondary objectives of our study included the following: (1) determining reasons for training failure, (2) reasons for early exit from HHD, and (3) timing of program exit as a function of reason for exit.

## Methods

### Study Design and Patients

This was a population-based, retrospective cohort study of AKC-S incident adult (≥18 years) patients on HHD who started their training between January 1, 2013—June 30, 2020 Incident patients were defined as individuals who have not previously been on HHD, and who underwent at least one day of training. The AKC-S HHD program started August 2004^3^ and currently we have 106 active patients. The majority, (66%) of patients, are treated with hemodialysis 5 to 6 times per week for a minimum of 5 hours (longer if dialyzing at night) with blood flow rate 250 mL/min and dialysate flow 300 mL/min while the remaining pursue conventional hemodialysis 3 to 4 hours at least 3 times per week with blood flow rate 300 mL/min and dialysate flow 500 mL/min. All patients use high flux dialyzers.

Training failure was defined as starting training but not succeeding to performing dialysis at home. Reasons for training failure were categorized as follows: (1) medical (unable to continue training due to appearance of a prohibiting medical condition, or progression of an existing medical condition); (2) psychosocial (socioeconomic factors hindering ability to complete training); (3) patient request (request made by patient to return to ICHD for a variety of reasons not defined as medical or psychosocial); (4) death (patient died during training); and (5) other (reason did not fit into 4 other sub-categories of training failure). Time on HHD was determined from the day of the first hemodialysis run at home until program exit (date of death, transplant, technique failure or study end date), this is considered time 0 for all outcomes of interest. As there is no consensus on the definition of technique failure in HHD, we ultimately defined technique failure as a transfer out of HHD with no return for greater than 60 days in order to avoid capturing temporary exits to ICHD. Like training failure, technique failure categories were also sub-categorized as follows: (1) medical (unable to continue training due to appearance of a prohibiting medical condition, or progression of an existing medical condition); (2) psychosocial (socioeconomic factors hindering ability to complete training); (3) safety/adherence concerns (formal concerns raised by patient’s family or kidney care team about their ability to safely dialyze at home; (4) moved (relocated such that they were unable to remain on the AKC-S HHD program; and (5) opted to change to PD. Early exit was defined as exit from HHD prior to 12 months. Approval was obtained from the University of Calgary Research Ethics Board for this study and informed consent was waived.

### Outcomes

The primary outcomes were time to exit and the reasons for exit from the HHD program (transplant, death, and technique failure reasons). Deaths were included if the patient died while on HHD or if they had exited the program and died within the 60 days of exit. Secondary outcomes included: reasons for training failure, reasons for early exit from HHD, the risk factors for technique failure and technique survival at 1 and 2 years.

### Data Sources

The data were gathered from the AKC-S electronic medical record (EMR) and included patient demographics, medical comorbidity, etiology of kidney failure, type of access at start of HHD (fistula vs central venous catheter [CVC]), training start date, first HHD session date, program exit date, and cited reasons for exiting the program. Categories of reasons for exit from HHD included death, transplant, technique failure (medical, psychosocial, safety, and relocation as defined above) and were determined after consultation with members of the care team and prior to documentation in the EMR. In cases where the exit reasons were not clearly documented, the HHD charge nurse and medical director reviewed the patient’s history again.

### Statistical Methods

The baseline characteristics were described by summary statistics at time of entry into the HHD program. We reported these summaries using means with standard deviation (SD) or median with inter-quartile range (IQR) for continuous variables and frequency and proportion for categorical variables. Comparisons between patients who exit or remain in the HHD program were assessed using two-sample t-tests and Wilcoxon rank sum tests for continuous variables and Fisher’s exact test and median unbiased estimate for binary variables. We used a Cox proportional hazard model to assess the association between patient characteristics including sex, age, type of vascular access, relationship status, comorbidities, and the outcome of technique failure. Exit from the HHD program was defined using three endpoints, where time 0 was identified as time of first HHD session in the patient’s home: (1) death, (2) transplant, and (3) technique failure where death and transplant were viewed as competing risks. Patients who stayed in the program were censored at the end of the study period. The cumulative probability of death, transplant and technique failure were determined using the Fine and Gray approach.^17^ The proportionality assumption for the competing risks regression was checked graphically for each predictor by plotting log (−log(F − 1)) against log (time). A two-sided *P*-value of <.05 was considered statistically significant.

## Results

**Figure 1.**
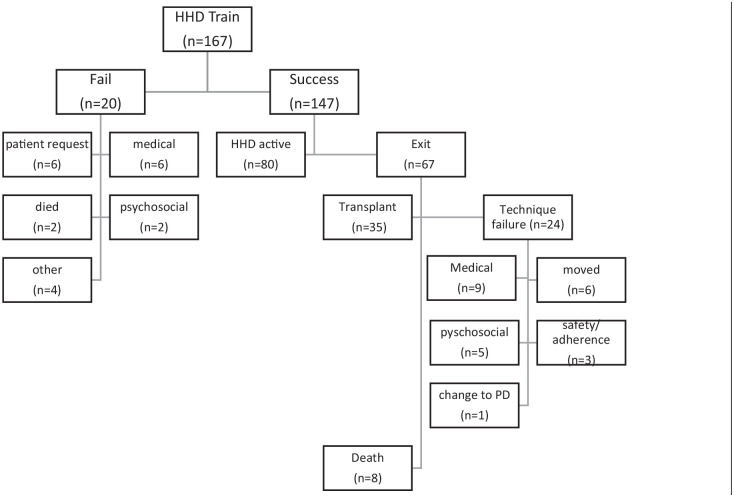
Disposition of patients enrolled in home hemodialysis. *Note.* HHD = home hemodialysis.

During our study period, a total of 167 patients entered the HHD program. Of this cohort, 68% of patients were male, with an average age of 57 ± 13 years and a dialysis vintage of 1.67 ± 3.1 years. The most common etiology of kidney disease was glomerulonephritis (29.3%) and the most frequent type of vascular access was a CVC (55.8%). The majority of participants were either married or in a common-law relationship (66.0%). Of the 167 patients who entered the program, 20 (12.0%) exited via training failure, while 147 (88.0%) patients successfully completed training (Figure 1). Table 1 shows the patient demographics for both the training failure population and patients that successfully completed training and entered the HHD program (underwent at least one session of dialysis at home).

**Table 1.** Patient Demographics.

Variable	Non-tech failure	Tech failure	Overall	*P*-value	Train failure	*P*-value
	n = 124	n = 24	n = 147			
Sex						
Male	83 (67.5%)	17 (70.8%)	100 (68.0%)		14 (70.0%)	
Female	40 (32.5%)	7 (29.2%)	47 (32.0%)	.93	6 (30.0%)	1.00
Age						
Mean (SD)	55.9 (12.4)	60.7(16.4)	56.7 (13.0)		64.4(10.9)	.007
Vascular access						
CVC	70 (56.9%)	12 (50%)	82 (55.8%)		12 (60.0%)	
AVF	53 (43.1%)	12 (50%)	65 (44.2%)	.54	8 (40.0%)	.91
Relationship status						
Single	29 (23.6%)	4 (16.7%)	33 (22.4%)		3 (15.0%)	
Married	72 (58.5%)	16 (66.7%)	88 (59.9%)		13 (65.0%)	
Common-law	8 (6.5%)	1 (4.2%)	9 (6.1%)		0 (0%)	
Unknown	14 (11.4%)	3 (12.5%)	17 (11.6%)	.89	4 (20.0%)	.56
ESRD etiology						
HTN	20 (16.3%)	3 (12.5%)	23 (15.6%)		9 (45.0%)	
DM	19 (15.4%)	8 (33.3%)	27 (18.4%)		1 (5.0%)	
RAS/ischemic	5 (4.1%)	1 (4.2%)	6 (4.1%)		1 (5.0%)	
GN	39 (31.7%)	4 (16.7%)	43 (29.3%)		3 (15.0%)	
PCKD	20 (16.3%)	2 (8.3%)	22 (15.0%)		1 (5.0%)	
Obstructive	2 (1.6%)	0 (0%)	2 (1.4%)		0 (0%)	
Other	14 (11.4%)	6 (25.0%)	20 (12.6%)		5 (25.0%)	
Unknown	4 (3.3%)	0 (0%)	4 (2.7%)	.22	0 (0%)	.05
Comorbidities						
CAD	23 (18.7%)	10 (41.7%)	33 (22.4%)	.02	6 (30%)	.57
DM	38 (30.9%)	11 (45.8%)	49 (33.3%)	.17	7 (35.0%)	1.00
CHF	17 (13.8%)	6 (25%)	23 (15.6%)	.19	9 (45.0%)	.004
PVD	13 (10.6%)	6 (25%)	19 (12.9%)	.08	1 (5.0%)	.47
CVD	10 (8.1%)	3 (12.5%)	13 (8.8%)	.50	5 (25.0%)	.045
Cancer	25 (20.3%)	4 (16.7%)	29 (19.7%)	.71	8 (40.0%)	.05
Chronic pulmonary	7 (5.7%)	5 (20.8%)	12 (8.2%)	.03	0 (0%)	.36
>2 comorbidities	74 (60.2%)	18 (75%)	92 (62.6%)	.18	19 (95.0%)	.009

*Note.* CVC = central venous catheter; AVF = arteriovenous fistula; ESRD = end-stage renal disease; HTN = hypertension; DM = diabetes mellitus; RAS = renal artery stenosis; GN = glomerulonephritis; PCKD = polycystic kidney disease; CHF = chronic heart failure; PVD = peripheral vascular disease; CVD = cardiovascular disease.

### Home HD Training

The total training time, median [inter-quartile range], for all was 6.0 weeks [4.0, 8.4] and 6.3 weeks [4.9, 8.4] after removing the patients who failed training (Table 2). Patients failed training at a median time of 3.1 weeks [2.0, 5.5]. The most common reasons for exit from the training program were patient preference and medical (see Table 3). Patients that failed training were more likely to be older (64.4 ± 11 years vs 56.7 ± 13 years, respectively, *P* = .007), have congestive heart failure (45.0% vs 15.6%, *P* = .004) or have two or more comorbidities (95.0% vs 62.6%, *P* = .009) as compared to those who successfully started on HHD (Table 1). The proportion of patients who were not married or common-law was similar (15.0% vs 22.4%, *P* = .56).

**Table 2.** Patient Outcomes.

Outcome	No. of patients n (%)	Train time median [IQR] (weeks)	Time on HHD median [IQR] (months)
Remaining on HHD	80 (47.9 %)	7.0 [5.1, 9.1]	25 [14, 46]
Transplant	35 (21%)	5.1 [4.2, 7.1]	17 [9.0, 32]
Death	8 (4.8%)	5.6 [5.4, 7.6]	32 [18, 46]
Technique failure	24 (14.4%)	6.1 [4.5, 8.9]	17 [8.9, 35]
Training failure	20 (12%)	3.1 [2.0, 5.5]	NA
All-comers	167 (100%)	6.0 [4.0, 8.4]	23 [11, 41]

*Note.* Time on HHD calculated only for the 147 patients who successfully completed training. IQR = inter-quartile range; HHD = home hemodialysis.

**Table 3.** Train Failure Reasons.

Exit reason (#)	Sub-categorization (#)	Train time median [IQR] (weeks)
Preference (6)	Excessive workload (2)	2.8 [1.1, 4.2]
	General weakness/lack of energy (1)	
	Close proximity to incentre dialysis location (1)	
	Lifestyle changes (1)	
	Undisclosed (1)	
Medical (6)	Pulmonary disease (1)	4.1 [3.1, 6.6]
	Chronic pain (1)	
	Access site infection (1)	
	Burns with resultant hospitalization (1)	
	Intradialytic GI upset (2)	
Death (2)	Sepsis (2)	1.9 [1.4, 2.3]
Psychosocial (2)	Loss of support system (1) Inability to cope with stress (1)	5.1 [3.6, 6.7
Other (4)	Safety concerns raised by care team (1)	3.9 [2.9, 5.1]
	Arthritis with dexterity issues (1)	
	Visual impairment (1)	
	Electrical inadequacy of home (1)	

*Note.* IQR = inter-quartile range.

### Home HD Outcomes

Of the 147 patients who successfully completed training, 80 (47.9%) remained in the program at the end of the study period. The primary outcome, median time to HHD exit, was 23 [11, 41] months, when excluding the patients that failed training and 21 [9.7, 37.4] when including the train failures (Table 2). The most common reasons for exit from HHD were transplant (35; 21%), followed by technique failure (24; 14.4%) and death (8; 4.8%). The median time to technique failure was 17 [8.9, 35] months from the first day of home HHD. Furthermore, 41.7% of the technique failure episodes occurred within the first 12 months. Patients who experienced technique failure were similar in age, gender and relationship status but a higher proportion had coronary artery disease, (CAD) (41.7% vs 18.7%, *P* = .02) and a chronic pulmonary condition (20.8% vs 5.7%, *P* = .03) as compared to those who did not experience technique failure (Table 4).

**Table 4.** Hazard Ratios for Technique Failure Using the Competing Risk Model.

Patient characteristic	Hazard ratio [95% CI]
Age	1.02 [0.98-1.06]
Access type	1.11 [0.50-2.43]
>2 comorbidities	2.05 [0.82-5.13]
CAD	2.67 [1.20-5.92]
DM	2.20 [1.00-4.80]
CHF	1.84 [0.73-4.66]
PVD	2.16 [0.91-5.14]
CVD	1.45 [0.44-4.77]
Cancer	0.80 [0.27-2.38]
Pulmonary disease	3.25 [1.17-9.03]

*Note.* CAD = coronary artery disease; DM = diabetes mellitus; CHF = chronic heart failure; PVD = peripheral vascular disease; CVD = cardiovascular disease.

The timing of exit from HHD was earlier for patients who experienced technique failure due to psychosocial reasons (median [IQR] = 13 [8.9, 18]) (Table 5). Examination of the 10 patients with early exit, as defined by leaving earlier than 12 months, revealed 3 patients left due to psychosocial reasons including loss of caregiver support, fear of loss of consciousness on dialysis due to intradialytic hypotension, and eviction from home. Two patients moved and switched to ICHD, one of whom moved to a different province. Four patients left due to medical reasons, specifically sepsis-related complications. One patient switched to PD at 5.7 months. Kidney transplantation occurred by 12 months of HHD in 12 (34%) of patients.

**Table 5.** Categorization of Technique Failure.

Exit reason (#)	Sub-categorization (#)	Train time median [IQR] (weeks)	Time on home hemodialysis medial [IQR] (months)
Medical (9)	Cognitive issues (2)	7.0 [5.4, 8.9]	18 [8.9, 51]
	Intradialytic hypotension (1)		
	Palliation due to comorbid medical condition (1)		
	SOB due to comorbid medical condition (1)		
	Sepsis and hospitalization (1)		
	Prolonged hospital admission with rehabilitation (1)		
	Intradialytic GI 1symptoms (1)		
	Undisclosed (1)		
Psychosocial (5)	Marital dissolution, loss of residence (1)	8.0 [5.1, 9.1]	13 [8.9, 18]
	Eviction (1)		
	Loss of support system (2)		
	Fear of AVF needling (1)		
Safety/adherence (3)	Cognitive issues (2)	5.4 [4.9, 8.9]	19 [15, 36]
	Lab testing non-adherence (1)		
Moved (6)	Interprovincial relocation (1)	5.6 [4.3, 8.2]	22 [9.1, 32]
	Intraprovincial relocation (2)		
	Relocated countries (2)		
	Undisclosed destination (1)		
Changed to PD (1)	Changed to PD (1)	4.3 [4.3, 4.3]	5.7 [5.7, 5.7]

Over the course of the study there were 8 deaths with the following causes: sepsis (2) (including 1 catheter-associated bacteremia); catastrophic complication of dialysis with resultant air embolus (1), pre-existing medical conditions not related to dialysis/kidney failure (2), seizure (1) and unknown (2). One of these deaths occurred before 12 months of HHD.

**Figure 2.**
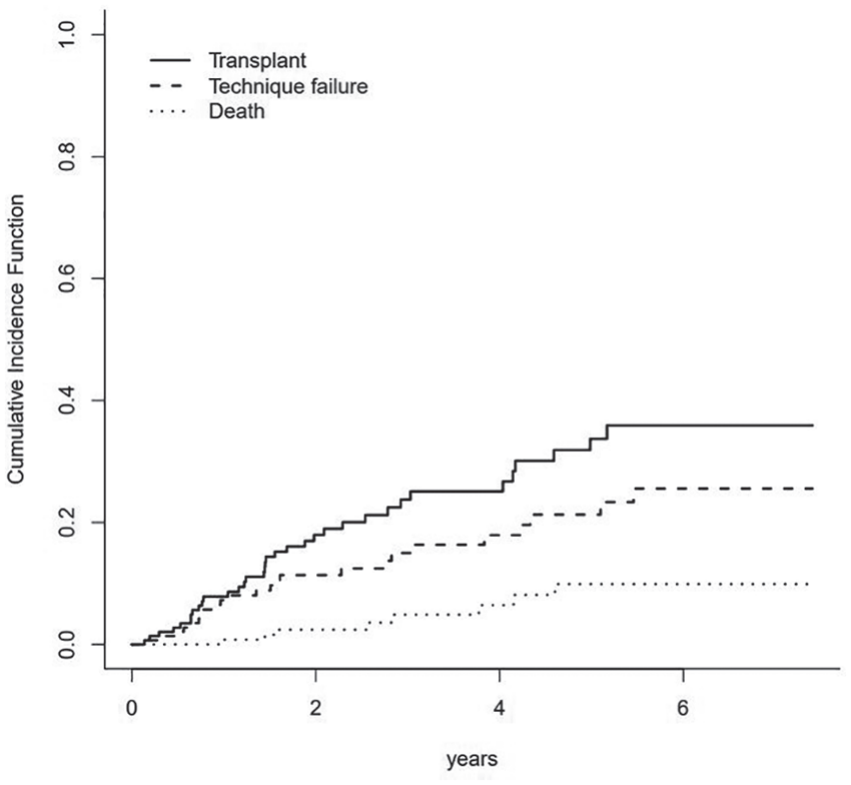
Competing risk curve for transplant, death, and technique failure.

In our HHD program, the overall technique survival rates were 92.7% at 1 year, 88.6% at 2 years, and 85.06% at 3 years. Figure 2 shows the cumulative incidence estimates for competing events of death, transplant and technique failure.

### Risk Factors for Technique Failure

We explored for risk factors of technique failure using the Fine-Gray regression model and found a history of coronary artery disease (CAD) (HR = 2.7, 95% CI = [1.20-5.9], *P* = .02), and diabetes (HR = 2.2, 95% CI = [1.0-4.8], *P* = .048) were associated with an increased risk of technique failure.

## Discussion

We describe a Canadian cohort of 167 incident patients on HHD in a single program who were followed from the onset of training and had a median time on HHD of 21 months. Amongst those who successfully completed training and went on to dialyze at home, the mean time on HHD was 23 [11, 41] months, exceeding the 12-month threshold to achieve cost savings with ICHD. Transplant was responsible for most of the exits from HHD at 21% (consistent with 8.2% to 30% in other studies)^14,18,19^ followed by technique failure at 14.4% (vs 10% to 25% reported by others).^14,15,18-20^

We found that those with comorbidities of CAD and DM were at an increased risk of technique failure, which align with findings from other HHD cohorts.^14,15^ Unlike others,^14,15,18,19^ we did not observe an association between age and technique failure (*P* = .17). In a similar study^15^ of 166 incident patients on HHD followed over 8 years, the risk of combined training and technique failure increased with older age, DM and degree of comorbidity. Brar et al have found that frailty is a predictor of technique failure in patients on home dialysis.^20^ As multi-morbidity in patients on HHD may reflect underlying frailty, it is possible that frailty is a contributor to technique failure in patients with comorbid conditions in our HHD program as well. This would be an important variable to consider in future work.

In reviewing the reasons for program exit, we found that the largest cause of technique failure was medical, followed by psychosocial, with safety and adherence concerns quite uncommon. Few studies report detailed reasons for HHD technique failure and given the lack of standardized reporting and inconsistences in the definition of “technique failure,” it is hard to compare our results to other studies. Trinh^19^ for example, did not report on the medical causes but had categories of other (21%) and unknown (23%) in addition to social (33%). Pauly^14^ had very similar technique failure rates (20% vs 14% in ours) and also found that medical reasons were a main reason of exit from their program (21%) followed by relocation (17%), loss of social support (17%) and inability to cope. While we followed the approach that Pauly et al took and considered relocation as a cause of tech failure, we recognize that other studies did not.^21^

Leaving HHD prior to 12.6 months, a time at which HHD achieves cost savings,^12^ is not optimal and HHD programs should aim to prevent or minimize these early exits. Trinh^19^ et al determined the rate of technique failure over time and found a higher rate in the first year. While they hypothesized that this is due to difficulties with the technical requirements of dialysis, they did not report the reasons for failure. Our study reports detailed reasons for HHD failure that correspond with timing of HHD exit, both important program metrics. We found that psychosocial reasons were an important driver of leaving early at a median time of 8.9 months, while safety (19 months) and medical concerns (26 months) appear later. We also observed a longer training time amongst the patients who developed technique failure due to psychosocial reasons: 8.0 [5,1, 9.1] vs 6.1 [4.5, 8.9] weeks. In our study, many of the psychosocial reasons for HHD exit revolved around the loss of social or personal supports highlighting the significant burden placed on family and support persons and the critical role that these supports play to maintain patients at home. These findings are consistent with patient and caregiver perspectives,^22^ while social isolation and a lack of peer support have been reported as factors affecting HHD patient decisions to leave in other studies.^19,22,23^

It would be important for the HHD team to assess the needs of patients who appear to be struggling in order to provide targeted interventions that meet their support needs. Increased support from the HHD program in terms of providing home visits, increased access to emotional and mental health supports, peer support and perhaps even a HHD assist program^24^ akin to what has been established in PD^25^ could help reduce some anxiety, prevent caregiver burnout and subsequent early exits and/or training failure from HHD. It may also be that the demands that a HHD program places on patients become onerous over time with increasing safety and adherence concerns manifesting later (19 months). Given these observations, perhaps programs should review their demands on patients with an eye to reduce the patient burden of care and workload. Examples of a reduced program ask could include the following: reduced frequency of blood work, outsourcing the dialysate and water sampling to technicians, reducing the number of in-person clinic visits in favor of virtual medicine, and reducing the frequency of audit requirements. Regular bi-annual vascular access audits in conjunction with re-training for patients at risk are important interventions that can reduce patient error^26,27^ and hopefully lead to a decrease in technique failure.

As seen by others,^14^ a significant number of patients (25%) exited HHD due to relocation as a cause of technique failure. An important component of the HHD screening process should be a thorough review for planned relocation in order to prevent this type of early exit. In our study, the median time spent on HHD prior to relocation was 22 [9.1, 32] months reflecting an effective screening process. Unfortunately, we did not track the primary motivation for relocation, or the incidence of continued HHD use upon relocation outside of our program, but the rationale behind these decisions could be instructive given the extensive training and significant time investment on HHD.

Death on HHD was only a small contributor to program exit (4.8%) and tended to occur later on in therapy (median: 32 months). Pauly et al^18^ also noted a low rate of death in their HHD patients (at 10% over 12 years) and attributed this to the fact that frail or failing patients on HHD at risk of dying are transferred to ICHD. While the death rate was low, we did have two patients die from sepsis from catheter related bacteremia and one catastrophic event leading to death, which is exceedingly rare based on reported events in the literature.^28^ Catheter related bacteremia on the other hand continues to be a significant cause of death on all forms of hemodialysis^29^ and might be modifiable in HHD with regular vascular access audits.^27^

Our study provided the opportunity to detail training failure rates, an important component of every HHD program. The finding of a 12% training failure rate is within the rates (2%-20%) reported by others,^14,16^ yet higher than that reported in another Canadian cohort (2%).^14^ In our study, the two most common reasons for training failure were patient preference (responsible for the majority of the early training exits), and medical reasons which occurred later in the training period. Although patients’ decision to leave an HHD training program earlier in the training process may be preferred from a resource standpoint, specifics surrounding these decisions should be documented clearly in order to circumvent modifiable factors for patient training exit. Regardless, education regarding the program expectations and lifestyle requirements remains critical during the patient screening period. Our rate of training losses for medical reasons (30%) was similar to Schachter’s et al.^16^ (21%). Both our study and that by Schachter^16^ et al highlight that patients and family may require additional psychosocial supports to prevent training failure. While it is difficult to make comparisons with such small numbers of patients, the differences in the rate of safety and adherence issues (5% vs 13%) may suggest a robust home dialysis eligibility assessment in our program, or it might indicate that our program requirements are too rigorous and result in increased disqualification from entry into HHD training.

Our study was conducted using data from a fairly large sample of patients on HHD as compared to other studies done in this area and provided a wide variety of detailed reasons for training and technique failure at different points during therapy. We recognize that it is difficult to pinpoint one single reason for program exit, given the complexity of patients with kidney failure, and the high likelihood that a multifactorial etiology is what often results in HHD patient loss. This highlights the challenge in prioritizing targeted interventions to improve therapy outcomes. Regardless, we have identified a number of areas that could begin to address many of these reasons for technique failure in hopes of reducing attrition rates and ultimately increasing the number of patients on home dialysis.

Though our sample was large compared to other HHD studies,^15,20^ with a relatively small patient population, it remains difficult to draw statistically significant conclusions regarding our primary and secondary outcomes. Another notable limitation was that our data was obtained retrospectively from EMRs, thus the quality of our data was dependent on the quality and availability of data within the EMR. The retrospective nature of our study also limits our ability to gain valuable insight into patient and caregiver perspective into the requirements of the training and performance of the HHD program. In addition, we relied on the documentation of the reasons for training and program exit which are often subjective and not standardized. These limitations coupled with practice difference across kidney programs may limit the generalizability of our findings.

## Conclusions

Home hemodialysis is a cost-effective modality that enhances patients’ quality of life in kidney failure treated with hemodialysis. However, significant turn-over occurs, and many patients do not successfully complete training. Of the patients who complete training and begin dialyzing at home, early exit due to technique failure remains a concern. We found that early technique failure occurred mostly due to psychosocial reasons, highlighting the need for increased social support as a potential modifier. In addition, patients with DM and CAD may be at increased risk of leaving the program. Interventions are required at the screening, training, and maintenance at home phases in order to reduce attrition and enhance success rates of patients on HHD. Future research that engages and collaborates with patients and caregivers is needed in order to identify and prioritize their needs to address the current gaps in care for patients on home dialysis.
